# Doxycycline: An essential tool for Alzheimer’s disease

**DOI:** 10.1016/j.biopha.2025.118159

**Published:** 2025-07

**Authors:** Gianluigi Forloni

**Affiliations:** Department of Neuroscience, Istituto di Ricerche Farmacologiche Mario Negri IRCCS, Italy

**Keywords:** Repurposing drug, Amyloid, Inflammation, Tetracyclines, Precision Medicine, Prion diseases

## Abstract

The identification of active interventions in neurodegenerative disorders is a major challenge in neurology; the use of repurposed drugs may represent a valuable strategy. Tetracyclines, a second generation of antibiotic molecules, offer various potential applications. Following an anecdotal observation of the potential anti-amyloidogenic activity of iododoxorubicin, the search for chemical analogs with a better safety profile led to tetracyclines. Their heterocyclic structures with a planar conformation interfere with b-sheet amyloid formation. Thus, doxycycline, a derivative with favorable blood-brain barrier penetration, emerged as a strong candidate to combat peripheral and central amyloidosis. In particular, we tested the anti-prion activity of doxycycline *in vitro* and *in vivo* experiments, confirming its capacity to disrupt or inhibit the formation of prion protein aggregates associated with pathological events. Treatment with doxycycline in human subjects with prion - related encephalopathies yielded contradictory results, suggesting that a preventive approach is a more favorable condition to verify efficacy; a clinical trial involving subjects at genetic risk of developing fatal familial insomnia, exposed to doxycycline for ten years, is currently ongoing. The anti-amyloidogenic capacity of doxycycline, combined with its safety profile in long-term treatment, has suggested its use in peripheral amyloidosis, which was tested with positive results. A specific interaction with β-amyloid or α-synuclein oligomers, as well as tau aggregation has also been demonstrated. More recently, the action of doxycycline has been extended to its anti-inflammatory and antioxidant capacities. In particular, the anti-inflammatory activity of doxycycline may explain the drug 's efficacy in numerous experimental models where protein misfolding has been associated with neuroinflammation, including Huntington's and Parkinson' s diseases. Thus, the pleiotropic action of doxycycline appears to be an interesting tool for addressing progressive neuronal dysfunction in multifactorial neurodegenerative diseases. The application of precision medicine principles to doxycycline treatment represents the best strategy to determine its efficacy. These aspects are illustrated here concerning another pleiotropic tetracycline, minocycline.

## Highlights

• Tetracyclines are a well-known class of antibiotics that exert several other pharmacological activities.
• Doxycycline and minocycline are two derivatives that pass the blood-brain barrier.
• The neuroprotective activities of these tetracyclines have been demonstrated in numerous investigations.
• Doxycycline exerts anti-inflammatory and anti-amyloidogenic activities.
• Doxycycline could be part of an innovative therapeutic strategy for Alzheimer's disease.

## Graphical Abstract

**Figure:**
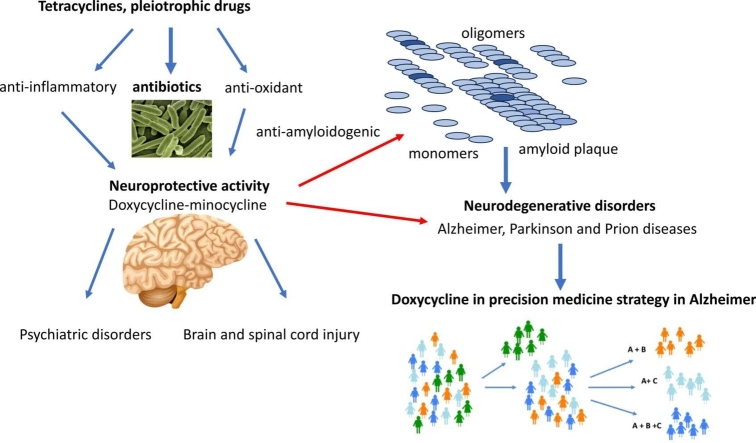

## 1 Introduction

**Fig. 1:**
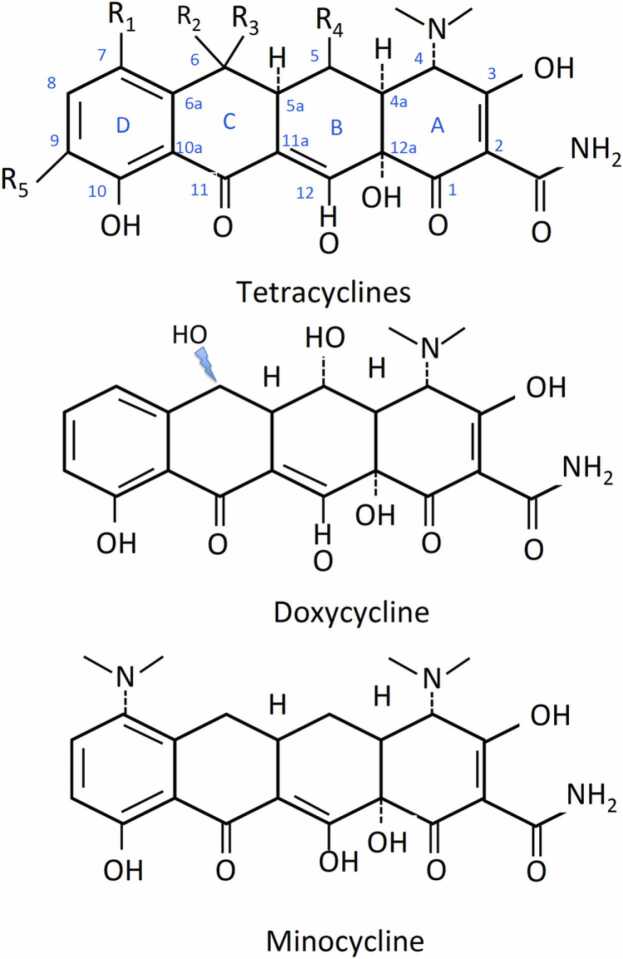
Chemical structure of tetracyclines.

**Fig. 2:**
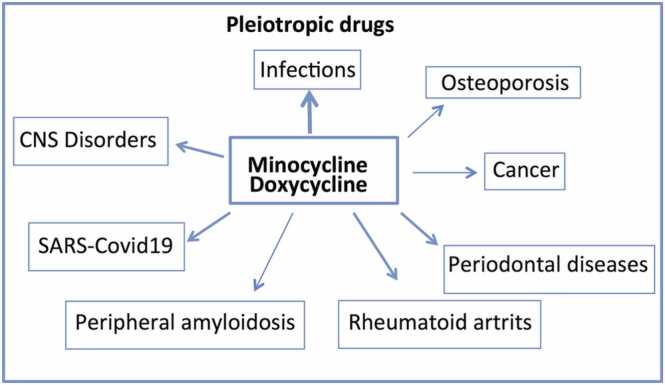
The broader spectrum of pharmacological activities for doxycycline and minocycline; the arrow thickness indicates the consolidation of experimental and clinical results supporting the pharmacological area of intervention.

Tetracyclines, initially discovered as natural compounds produced by *Streptomyces aureofaciens* [1], [2], constitute an important class of broad-spectrum antibiotics that inhibit bacterial growth by interfering with protein biosynthesis. The fundamental structure of tetracyclines consists of four linearly condensed benzene rings in a hydronaphthacene nucleus. The substituents at the C5, C6, C7, and C9 positions characterize the analogs (Fig. 1). The first generation of tetracyclines, including tetracycline itself, was discovered in the 1950s; tetracycline was derived from the catalytic hydrogenation of chlortetracycline. Tetracycline exhibits better pharmacokinetics, promoting its extensive therapeutic use. The second generation of tetracyclines, obtained by semi-synthesis, was optimized in terms of antibacterial activity, pharmacokinetics, toxicology, and blood-brain barrier penetration. The main representatives of this class were doxycycline and minocycline [3], [4]. A third generation of tetracyclines has been more recently developed to enhance potency and expand the spectrum of activity against bacteria resistant to other tetracyclines. After three decades, a new tetracycline has been approved for clinical use: tigecycline, a synthetic derivative of minocycline [5], [6]. It represents a prototype of this new generation, offering superior potency against Gram-positive and Gram-negative multidrug-resistant bacteria [7]. Although other synthetic approaches to overcome bacterial resistance are under investigation [8], the development of new tetracycline derivatives to address TDase-mediated resistance has been recently proposed to restore antibacterial activity [9]. The antimicrobial activity of tetracyclines has been associated in the last two decades with additional effects observed in various pathological conditions [10], [11], [12], [13], [14], [15], including COVID-19 infection [16], [17]. The potential antiviral activity associated with antibiotics, including tetracyclines, has been recently investigated with positive indications; however, appropriate clinical trials are recommended to define the effective antiviral activity of these antibiotics [18]. Independently of their antimicrobial activity, the anti-inflammatory properties of tetracyclines have been demonstrated in different pathological conditions [19], [20], and their use has recently been confirmed as part of a strategy to control chronic inflammation [21], [22]. The anti-inflammatory and immunomodulatory activities of tetracyclines, particularly in doxycycline and minocycline, have been recently linked with anti-apoptotic, antioxidant, and anti-protein aggregation properties to support their utilization in neurological and psychiatric fields [23], [24]. Tetracyclines have been proposed with curative efficacy in different neurological conditions, both at experimental and clinical levels. In particular, the repurposing of doxycycline in neurology is supported by abundant results [25], [26], [27], [28], [29], [30] and requires innovative approaches using this drug to develop effective therapeutic strategies.Fig. 2

## 2 Tetracyclines: pleiotropic drugs

### 2.1 The anti - microbial activity

The first tetracycline, aureomycin, was discovered in 1948. By analyzing numerous soil samples sent from different parts of the world, it was observed that actinomycete bacteria produced a yellow colony with a remarkable inhibitory effect against many pathogenic strains, including rickettsia and Gram-positive bacteria [1]. In similar investigations, which tested over one hundred thousand soil samples in search of new compounds, Finlay et al. (1950) [31] discovered a soil bacterium, Streptomyces rimosus, that also produced a golden active compound initially named terramycin. The structures of aureomycin and terramycin were identified as chlorotetracycline and oxytetracycline [32], and NMR and crystallographic analyses definitively clarified the structures ten years later [33], [34]. Tetracyclines have played an essential role in treating bacterial infections [2]. The bioavailability and pharmacology of tetracyclines are characterized by the formation of ionic and zwitterionic species due to the structural presence of vinylogous acid and a keto-enol system at the A, B, and C rings, respectively [35] (Fig. 1). On the other hand, acidic or basic conditions not only lead to the protonation/deprotonation of the amino and hydroxy groups but also to irreversible degradation reactions, as will be outlined later [36]. The efficient binding at various ribosomal binding sites results from the formation of metal complexes within the cells. The antibacterial activity of tetracyclines is based on their inhibition of protein biosynthesis in the target pathogens. They tightly bind to the 16S rRNA site on the 30S ribosomal subunit during translation, preventing the binding of aminoacyl-tRNA to the bacterial ribosome. This interference inhibits the entry of aminoacyl-tRNA into the acceptor site (A) on the ribosome, disrupting the incorporation of amino acid residues during polypeptide chain formation. Consequently, bacterial protein synthesis is halted, leading to the inhibition of bacterial growth [37]. The mechanism of action of tetracyclines is generally bacteriostatic because the interaction between tetracyclines and ribosomes is reversible; however, the new tetracyclines also exert bactericidal activity in in vitro tests [24]. Tetracyclines additionally bind to eukaryotic ribosomes, but with an affinity approximately fifteen-fold lower than that of bacterial ribosomes [38], [39]. Furthermore, significant differences exist in tetracycline uptake, making eukaryotic cells less susceptible to these antibiotics [40]. The possibility of reductively removing the hydroxyl group at C6 while retaining broad antibacterial activity in a more stable structure, 6-deoxytetracyclines, was the key discovery that opened the development of the second generation of tetracyclines [41], [42]. Both doxycycline and minocycline belong to this group of tetracyclines; doxycycline was initially synthesized from oxytetracycline in the mid-sixties [43], while minocycline, characterized by the presence of two nitrate groups, was discovered several years later [44]. The main advantage of minocycline is its broader spectrum of activity compared to previous derivatives, along with a better pharmacokinetic profile; it was the last tetracycline introduced to the market in the following three decades. The growing occurrence of bacterial resistance has stimulated scientists' interest in the development of new tetracyclines. The primary representative of this new generation of compounds is tigecycline, a synthetic derivative of minocycline, introduced into therapy after more than 30 years [45], [46]. Tigecycline has been widely used for treating bacterial infections caused by multidrug-resistant bacteria for which treatment options are limited [47], [48]. However, the rising incidence of tigecycline resistance, especially among Gram-negative bacteria, has become a significant issue that needs to be addressed through adequate studies on the resistance mechanisms to provide insights for drug development [49], [50]. Thus, other tetracycline derivatives have recently been approved by regulatory authorities due to their increased potency and efficacy, even against bacteria resistant to tetracycline [5], [8].

### 2.2 Other activities

The second-generation tetracyclines have a similar chemical structure and antibacterial spectrum. However, when applied in a non-antibiotic context, they exhibit different activities. It has been demonstrated that many cancer cell lines respond to treatment with tetracyclines by suppressing matrix metalloproteinases and promoting apoptosis [51]. The anti-inflammatory and antioxidant actions of minocycline, along with several other biological activities such as inhibition of mitochondrial activity and regulation of apoptotic mechanisms, have been recognized in anti-cancer therapy [52], [53]. However, minocycline exerts other functions potentially relevant to counteracting tumor growth, either individually or as an adjunct, particularly in the treatment of cerebral tumors due to its favorable passage across the blood-brain barrier [54], [55]. In the neuropsychiatric area, minocycline exhibits protective effects against various brain insults; its positive effects in neurological and psychiatric conditions are mainly explained by its anti-inflammatory activity [56], [57]. The efficacy of minocycline in controlling depressive symptoms in humans has been confirmed by a meta-analysis of randomized, double-blind, placebo-controlled trials [58], [59].

Although the positive effects of minocycline treatment have been demonstrated in numerous experimental models of neurological diseases, the results at the clinical stage are less favorable. In the Alzheimer's disease (AD) model, treatment with minocycline was originally proposed by Hunter et al. [60]; the drug reduced the impact of the p75-saporin lesion on basal forebrain cholinergic cells. Several other data have accumulated to support minocycline as a promising candidate for a therapeutic strategy in AD [61], [62], [63]. In addition to its anti-inflammatory activity, minocycline has been shown to directly interfere with tau and beta-amyloid aggregation in in vivo models [64]. However, in a randomized clinical trial (MADE), where two doses of minocycline were tested against placebo in mild AD, after 24 months of treatment, there was no difference in cognitive performance between the groups receiving minocycline or placebo [65].

Although the clinical outcomes were not encouraging, minocycline was subsequently tested in other experimental models of AD. Using a rat model of sporadic AD, Abdo Qaid et al. [66] demonstrated that minocycline antagonized the increase in tau phosphorylation induced by peripheral lipopolysaccharide (LPS) injection. Although tau hyperphosphorylation is associated with AD pathogenesis, in this model, the efficacy of minocycline relates to general anti-inflammatory activity rather than specific interference with tau pathology. Similar findings were noted in another sporadic AD model using streptozotocin injection, where the tetracycline alleviated cognitive impairment and some inflammatory markers [67], [68]. The neuropathological and behavioral damage induced by intracerebral application of Aβ in rats was prevented by chronic oral treatment with minocycline [69], [70]. In transgenic APP/PS1 mice, early treatment with minocycline reduced the microgliosis that appeared prior to cerebral plaque formation and antagonized cognitive behavioral deficits [71]. This interesting observation aligns with our results in a different transgenic mouse model of AD (Tg2576), where cognitive impairment preceded plaque formation and was neutralized by a modulator of γ-secretase [72]. Recently, Giraldo-Berrio et al. [73] investigated the interference of minocycline with different aspects of the pathophysiology of AD, including acetylcholine release, tau and β-amyloid aggregation, in cholinergic-like neurons derived from mesenchymal cells with and without a presenilin 1 mutation associated with AD. Taken together, these results seem to encourage new investigations with minocycline in AD subjects and, on the other hand, they highlight the problematic relationship between preclinical positive evidence and the challenges of confirming the results in clinical trials. The translational significance of the experimental results is limited by numerous factors, including the capacity of animal models to recapitulate pathological conditions, especially in aging-associated disorders, the intraindividual variability of pathological expression in humans regarding etiology and disease manifestation, and the difficulties in choosing the appropriate timing for treatment in the early phases of the disease according to the pharmacokinetic studies and pathogenetic development. However, as we discuss below, the main problem is the complexity of the disease, which requires a multifunctional approach and an accurate characterization of the AD subjects. However in the case of minocycline a treatment with the drug has been associated with some cognitive improvement in human healthy subjects [74].

In experimental models, minocycline consistently attenuates the consequences of traumatic brain injury (TBI) [75], [76]. In a recent paper, this positive effect has been linked to a reduction in aquaporin-4 levels, an improvement in blood-brain barrier (BBB) integrity, and enhanced astrocyte function [77]. However, a clinical evaluation has shown that treatment with minocycline reduced microglial activation but increased markers of neurodegeneration [78]. A subsequent pilot trial in TBI patients yielded more positive results and did not confirm the NFL reduction [79]. More recently, positive evidence has been cited in favor of tetracycline treatment in TBI [80], [81]. Neuroprotective effects of minocycline were observed in the treatment of spinal cord injury (SCI) [82], [83], and the anti-inflammatory activity was considered the principal driver of the positive effects of tetracyclines. We demonstrated that the local administration of minocycline through nanovectors capable of selectively targeting microglia effectively modulates resident microglial cells, reducing the pro-inflammatory response and improving behavioral outcomes in an SCI animal model [84], [85]. A similar approach has been proposed with the preparation of a minocycline polymer micelle thermosensitive gel, which, when applied in a rat SCI model, induced a neuroprotective effect [86]. In humans, the efficacy of minocycline in reducing the consequences of SCI was modest [87], [88], [89], but further investigations will be necessary to draw final conclusions on minocycline's activity in SCI [90], [91], [92], [93].

### 2.3 Doxycycline and neurodegenerative disorders

**Fig. 3:**
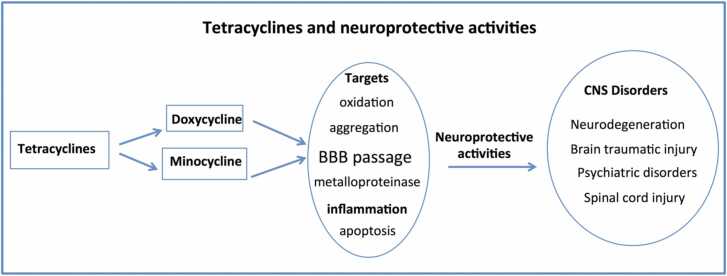
The diagram illustrates the common pharmacological targets and characteristics of minocycline and doxycycline that are important for the neuroprotective effects in CNS disorders.

**Fig. 4:**
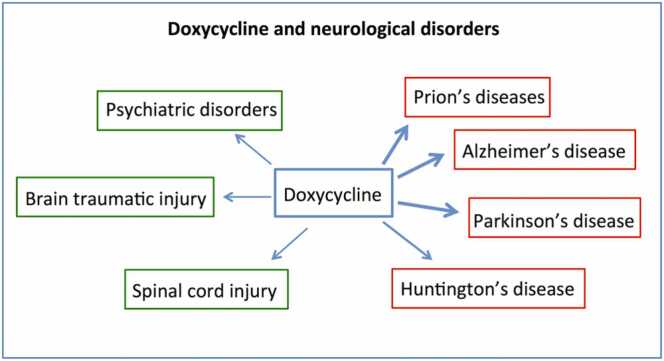
The doxycycline neuroprotective activities; the arrow thickness indicates the consolidation of experimental and clinical results in favor of therapeutic activities.

Although several non-antibiotic effects, including anti-inflammatory, antioxidant, and neuroprotective properties, are considered common features of both minocycline and doxycycline (Fig. 3) other aspects distinguish the activities of the two derivatives. As mentioned above, the efficacy of minocycline in a psychiatric context as an adjuvant to antidepressant therapy has been demonstrated in experimental models and substantially confirmed at the clinical level. In contrast, the antidepressant activity of doxycycline has been poorly investigated, and the evidence at the experimental level is limited to specific models, such as depressive-like behavior in mice following LPS injection, where inflammation may be more prominent than psychiatric symptoms. Other anecdotal cases [94] or isolated observations in experimental studies [95], [96] require more consistent data to support doxycycline as part of a therapeutic strategy for specific psychiatric conditions.Fig. 4

On the neurological side, the efficacy of doxycycline treatment has been well investigated in numerous pathological conditions; in addition to their anti-inflammatory and antioxidant activities, tetracyclines are characterized by their anti-amyloidogenic action [97], [98], [99], [100], [101]. The amyloidogenic process involves the polymerization of peptides into conformers that are rich in beta-sheets, which exhibit insolubility and a fibrillogenic ultrastructure, marking it as a fundamental pathological occurrence in AD and various protein misfolding disorders. The development of amyloid is characterized by shifts from oligomers to protofibrils and eventually to mature fibrils, following the seeding mechanism first introduced by Jarrett and Lansbury [102] and specified by Come et al. [103]. Several elements, such as pH, ionic strength, temperature, amino acid sequence mutations, metal presence, concentration, and incubation duration, influence amyloidogenesis [104]. The interference with the self-assembling capacity of amyloidogenic peptides can be assessed by appropriate methods; doxycycline at micromolar concentrations inhibited the formation of prion and beta-amyloid fibrils [97], [98], and, more recently, this capacity has been extended to tau and alpha-synuclein aggregation [105], [106], [107].

Doxycycline has been investigated in AD models with positive results [108], [109]. We showed that systemic treatment with doxycycline antagonized the harmful effects of the intracerebral application of Aβ oligomers on memory impairment and glial reactivity [108]. In APP/PS1 transgenic mice, acute and chronic treatment with doxycycline exhibited a beneficial effect on short-term memory; although only after prolonged exposure, this effect was associated with a reduction in Aβ plaques [108]. These results were consistent with direct interference with Aβ oligomers by doxycycline; however, the drug antagonized the LPS-induced cognitive decline in the absence of Aβ oligomers, confirming its anti-inflammatory activity [110], [111], [112], [113], [114]. Based on the antibiotic properties of doxycycline, two clinical trials were conducted in subjects with mild to moderate AD; positive findings from the first study [115] were not confirmed in the second trial, where doxycycline and rifampin, after 12 months of treatment, did not show, alone or in combination with rifampin, beneficial effects on cognition or function in AD [116]. Also, in this case, as reported for the minocycline trial in AD, the difficulties in translating positive evidence from preclinical studies to the clinical stage are part of the complexity of developing an effective strategy for AD.

### 2.4 Doxycycline and prion diseases

The chemical analogy with other molecules interfering with the self-assembling capacity of amyloid proteins has indicated tetracyclines as a possible candidate to exert anti-amyloidogenic activity. Following an anecdotal observation of potential anti-amyloidogenic activity of iododoxorubicin (IDX) [117], we tested the activity of this well-known anti-cancer drug in experimental scrapie, a model of prion diseases. Syrian hamsters were inoculated intracerebrally with either scrapie-infected brain homogenate or homogenate coincubated with IDX. In IDX-treated hamsters, clinical signs of disease were delayed, and survival time was prolonged [118]. This positive effect must be weighed against the fact that IDX 's toxicity does not recommend its use for chronic treatment; thus, we began the search for chemical analogs with a better safety profile, and the polycyclic planar structure of tetracyclines was immediately considered. Tetracyclines were positively tested in vitro, inhibiting the self-aggregation of prion peptide analogs to different fragments of the prion protein and reversing the protease resistance of the disease-associated prion protein isoforms extracted from the brain tissue of patients [119]. In vivo, tetracyclines substantially replicated the anti-prion activity of IDX in Syrian hamsters inoculated with infected scrapie brain homogenate [120]. Subsequently, it has been shown that systemic treatment with doxycycline also reproduced the same results [121].

The repurposing of doxycycline for prion disease has been examined in an observational study in which the drug was offered as compassionate treatment to patients diagnosed with Creutzfeldt-Jakob disease (CJD). Subsequent analysis of the results showed that CJD patients receiving doxycycline lived significantly longer compared to the untreated group [122]. Similar results were later obtained in an independent observational study in Germany [123]. On this basis, we designed the first randomized, double-blind, placebo-controlled trial in Creutzfeldt-Jakob subjects to test the efficacy of doxycycline. The results were negative, likely due to the advanced stage of the disease in recruited subjects [124]. This did not rule out the possibility of testing doxycycline's efficacy in a preventive trial; thus, we treated asymptomatic subjects carrying a mutation associated with fatal familial insomnia, a genetic prion disease. The disease is so aggressive that halting its progression at the onset was extremely unlikely, and prolonging survival in disease conditions was not desirable either. A preventive approach was preferable, which, if effective, could prolong health or even hinder the development of the disorder. The best possible protocol was developed based on historical data and the incidence of the disease during an observational period of ten years. The trial has recently concluded, and the results are under evaluation [125]. Regardless of the final outcome, several achievements have been made with this long-lasting study; for instance, we established the good tolerability of chronic treatment with doxycycline and observed no relevant side effects with a dosage of 100 or 200 mg daily. Furthermore, although the number of subjects enrolled was minimal, the statistical evaluation of disease incidence compared to historical data should provide sufficiently robust indications to consider treatment with doxycycline beneficial in the pre-symptomatic phase of the disease [126]. In the absence of appropriate biological markers in asymptomatic conditions, we had to evaluate the incidence of the disease over a long observation period. The availability of biological fluids collected longitudinally during the ten years of observation may prove to be an important source for identifying potential biological markers useful for monitoring the efficacy of the drug and the approach of disease onset [127].

### 2.5 The anti-inflammatory activity of doxycycline

Inflammation plays a role in the development of almost all neurodegenerative diseases. Initially viewed as a result of neuronal cell death, glial activation has increasingly been recognized as a key element during the early stages of these diseases when neuronal dysfunction begins [128], [129], [130], [131]. The beneficial role of surveillance in removing debris and harmful agents, performed through the phagocytosis by activated microglia and astrocytes, may, under certain conditions, be linked to the generation of factors that adversely affect the neuronal system. Continuous activation of glial cells that interferes with neuronal activity may result from persistent exposure to inflammatory stimuli or a breakdown in the resolution process [132]. Both mechanisms are important in AD and other neurodegenerative disorders, leading to persistent chronic inflammation [132]. The development of doxycycline as a therapeutic tool in prion diseases has been based on the anti-amyloidogenic effect of tetracyclines; however, during the last decade, several studies have highlighted other activities beyond antibiotic action, including anti-inflammatory capacity. The inflammasome-caspase-1 pathway plays a role in the onset of acute respiratory distress syndrome (ARDS), a diverse condition associated with elevated mortality rates. Tetracycline has been effectively utilized to treat individuals with direct ARDS, in which inflammation driven by IL-1β and IL-18 plays a significant role in the condition [16]. The first evidence of the anti-apoptotic and anti-inflammatory activity of doxycycline in a neurological context was presented by Choi et al. [133], who demonstrated the neuroprotective activity of doxycycline both in cultured dopaminergic cells exposed to LPS and in vivo following MPTP treatment. Successively numerous other studies confirmed the neuroprotective activity of doxycycline mainly mediated by anti-inflammatory activities [134], [135], [136]. In transgenic mice overexpressing the mutated form of synuclein associated with PD (A53T), we confirmed this perspective, highlighting both anti-amyloidogenic and anti-inflammatory activity as essential in countering the pathological spread in PD [137], [138].

### 2.6 Precision medicine approach

The repurposing of doxycycline in neurodegenerative disorders, particularly in AD, requires an innovative approach. The drug is well-tolerated, with minimal side effects, presenting a great opportunity to address some of the numerous difficulties encountered when designing clinical trials in AD appropriately. The translation to the clinical level of positive results derived from experimental studies is one of the main challenges in the field. Over the last three decades, knowledge about the pathogenesis of neurodegenerative diseases has grown tremendously. From molecular genetics to epidemiology, technological and methodological advances have been extraordinary; however, despite these efforts, these achievements still have limited effects on therapeutic strategies. The reasons for the numerous failures in finding disease-modifying treatments are varied; the complexities of the disease, including a long-lasting preclinical phase, and the absence of reliable surrogate markers to evaluate treatment efficacy are probably the most significant limitations, along with inappropriate patient selection. However, some of these issues, such as patient selection and the absence of biological markers, have been partially addressed in the recent positive studies with anti-Aβ antibodies, lecanemab, and donanemab [139], [140]. As disease-modifying drugs targeting β-amyloid in Alzheimer's disease emerged after two decades of failures and a quarter of a century following the first experimental evidence [141], they must be considered from the perspective of clinical practice [142]. Although the importance of these findings should not be underestimated, several aspects require clarification to evaluate their impact on clinical practice [143], [144], [145]. The narrow pathway between modest efficacy on one side and the presence of significant undesirable effects on the other, along with costs and the impact on healthcare system sustainability, will take time to reach a final opinion on the practicality of these treatments [146], [147], [148], [149], [150]. For instance, the accurate selection proposed for the patients recruited in the donanemab trials aligns with the study design, but it will be challenging to reproduce in routine clinical settings. According to the evaluation in the TRAILBLAZER-ALZ2 trial with donanemab, patient recruitment was conducted through PET analysis to assess β-amyloid and pTau deposition in the brain. Patients with very low levels of cerebral pTau deposition were considered ineligible for treatment, while others were stratified into low, medium, or high pTau deposition, which directly impacted treatment efficacy [140]. Furthermore, donanemab was discontinued in patients whose cerebral β amyloid deposits were less than 80 %, as determined by PET analysis. Several CSF or plasma markers, including NFL, GFAP, and pTau217 [151], [152], are now proposed for the diagnostic process and monitoring of drug efficacy, but the complete replacement of PET analysis in this context requires further confirmation. According to Cummings et al. [153], the number of subjects eligible for antibody treatment is limited, as observed by Logroscino et al. [154] in an Italian clinical setting. Thus, the introduction of the first disease-modifying treatments into clinical practice necessitates overcoming many difficulties, and even when all conditions are controlled, the efficacy of the antibodies does not appear definitive. However, after numerous attempts, it is now possible to clear the brain of β-amyloid deposits, providing a good starting point for more elaborate approaches to the disease. The complex nature of AD necessitates the use of multitargeted therapies; a therapy based on a single treatment is likely overly simplistic, if not incorrect, or at the very least insufficient. Therefore, adhering to the principle of precision medicine, the effectiveness of doxycycline for individuals at risk of developing AD could be explored alongside other neuroprotective therapies, but only after careful selection of the trial participants. As demonstrated here, doxycycline's anti-amyloidogenic properties are linked to its anti-inflammatory effects. Considering this evidence, the selection of individuals to be administered doxycycline should be based on their potential responsiveness to these effects. Indeed, the role of inflammation in the initial stages of AD development may vary both individually and over time among different patients. Based on the concept of personalized biomarker-driven targeted treatments, a substantial group of people experiencing mild cognitive impairment (MCI), including those who report subjective cognitive decline or even cognitively healthy individuals with additional risk factors for developing AD, should be assessed on different levels [155]. The MCI subjects eligible for multiple treatments must be selected through progressive steps of examination, employing neuropsychological tests, biochemical analyses in biological fluids, imaging when possible, and a final more specific characterization of the inflammatory profile . Along these lines, Lista et al. [156] recently proposed tracking the inflammatory markers (GFAP, YKL-40, and sTREM2 in plasma and CSF) in AD patients to enhance therapeutic strategies. Alongside inflammation, the heterogeneity of the cognitively impaired population must be investigated to select different profiles; multimodal investigations integrated with artificial intelligence-based methodologies [157], [158] must be determined to create additional profiles beyond inflammation, such as vascular [159], [160] or mitochondrial [161], [162], [163], [164], [165], to predict different disease trajectories and possible responders at various components of the therapeutic strategy

**Fig. 5:**
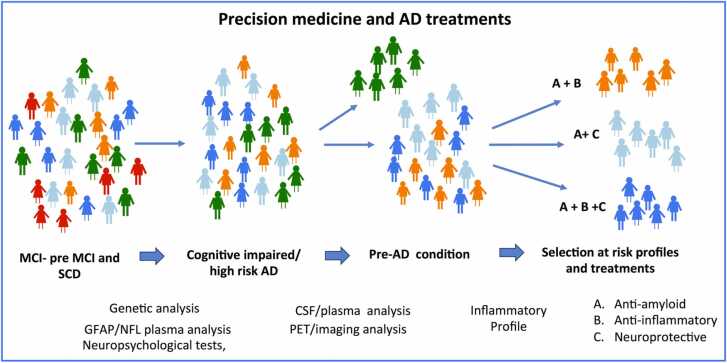
The diagnostic process to progressively identified candidates to a combination of treatments.

A similar approach has been proposed by Li et al. [166] using an AI-driven methodology to identify the best pharmacological strategy. In the revised criteria for diagnosis and staging of AD, proposed by the Alzheimer's Association in a recent update [152], AD is defined exclusively on the basis of biological markers. According to this view, AD initiates with the appearance of neuropathological changes, even in the absence of clinical evidence. The definition of AD, exclusively based on biological evolution, is discussed by another International Working Group [167], which considered asymptomatic individuals with biological patterns compatible with AD as subjects at risk; however, in the absence of symptoms, they cannot be considered AD patients [168]. Regardless of these diverse interpretations, the diagnostic process is based on a panel of biological markers in plasma (Aβ-42, GFAP, NFL, p-tau 217/181/231), imaging analysis (FDG, amyloid, and tau PET analysis), and neuropsychological examinations. Although GFAP in plasma can be considered an inflammatory marker, a more accurate selection of immunological parameters should be incorporated to develop inflammatory profiles and consequent personalized therapy. The determination of inflammatory cytokines in CSF significantly improves the prediction of conversion from control to MCI and from MCI to AD [169]. Apparently, CSF cytokines serve as prognostic indicators of cognitive decline. Genetic predisposition to higher circulating levels of IL-6 is associated with an increased risk for AD, as shown by analyzing a large cohort in a population-based study [170]. A similar indication comes from Abbatecola et al. [171], which suggests, through a systematic review, that determining circulating biomarkers of inflammaging is essential for improving diagnosis and dementia trajectories. Longitudinal analysis of gut microbiota will be useful in evaluating possible positive effects of treatment with doxycycline [172]. However, regardless of the prognostic value of the inflammatory profile, stratifying subjects on this basis should guide the therapeutic approach involving anti-inflammatory drugs like doxycycline [173], [174]. As illustrated in Fig. 5 the progressive steps of patient selection would be necessary to develop an appropriate therapeutic strategy. The multitarget activity of doxycycline can be tested in combination with other drugs, including neuroprotective agents and/or anti-Aβ antibodies that do not replicate a similar mechanism of action [155]. Using doxycycline in specific conditions can easily overcome most of the inconveniences caused by combination therapy. According to EMA guidelines, a treatment involving multiple drugs does not require a comparison with each individual treatment; instead, the combination can be compared directly to a placebo in a two-arm design [175], [176]. An alternative strategy is proposed by Cummings et al. [177], which includes add-on strategies involving an approved agent plus a new molecular entity. More recently, Cummings et al. (2025) addressed numerous aspects associated with combination therapy in AD, for instance, an anti-Aβ antibody plus another drug [178]. These recommendations must be carefully evaluated; for instance, the well-known pharmacodynamic/pharmacokinetic profile of doxycycline might easily support its use in a disease-modifying combination therapy.

Doxycycline is not a unique example of a drug with multiple activities that can contribute to developing an efficient therapeutic strategy in AD and other neurodegenerative disorders when combined with other agents that have diverse mechanisms of action. In prion diseases, small molecule inhibitors of SERPINA3/SerpinA3n, a serine protease inhibitor, promote prion clearance in chronically infected neuronal cells without altering PrPC metabolism or directly binding to PrPSc aggregates [179], [180]. This non-PrP-targeted strategy not only proved effective across different prion strains but also exhibited synergy when combined with quinacrine, a direct anti-prion drug, suggesting broader utility for combination treatments in prion and prion-like disorders [181]. These findings reinforce the need for therapeutic approaches that integrate multi-target drug design, aligning with the rationale behind doxycycline repurposing and its use in combinatory regimens aimed at modulating neuroinflammation, oxidative stress, and proteostasis mechanisms.

## 3 Conclusions

The pleiotropic activity of tetracycline has been utilized in various pathological conditions, and the repurposing of doxycycline and minocycline for neurological and psychiatric disorders is supported by numerous studies. Although the clinical data are less favorable than the experimental evidence, the anti-inflammatory and antioxidant properties of both minocycline and doxycycline suggest that these molecules are valuable pharmacological tools. In particular, we focusedour attention on doxycycline, a well-tolerated drug for chronic treatment with optimal penetration across the blood-brain barrier as proved by Lucchetti et al., [182]. The multi-target activity of doxycycline - especially its combination of anti-amyloidogenic and anti-inflammatory capabilities - positions this molecule as an important component of a therapeutic strategy in AD. The principle of precision medicine, when applied appropriately, can select subjects with cognitive decline in a pre-Alzheimer state combined with an "inflammatory profile," indicating the best candidates to test the efficacy of doxycycline alone or in combination with another neuroprotective drug. The urgency for effective treatments in AD strongly supports this innovative approach to mitigate some obstacles that have hindered the identification of active disease-modifying strategies.

## CRediT authorship contribution statement

**Gianluigi Forloni:** Writing – review & editing, Writing – original draft, Funding acquisition.

## Declaration of Competing Interest

The authors declare the following financial interests/personal relationships which may be considered as potential competing interests: Gianluigi Forloni reports financial support was provided by Telethon Foundation. If there are other authors, they declare that they have no known competing financial interests or personal relationships that could have appeared to influence the work reported in this paper.

## References

[1] Duggar B.M. (1948). Aureomycin: a product of the continuing search for new antibiotics. Ann. NY Acad. Sci..

[2] Ramachanderan R., Schaefer B. (2021). Tetracycline antibiotics. Chem. Texts.

[3] Saivin S., Houin G. (1988). Clinical pharmacokinetics of doxycycline and minocycline. Clin. Pharm..

[4] Cunha B.A., Baron J., Cunha C.B. (2018). Similarities and differences between doxycycline and minocycline: clinical and antimicrobial stewardship considerations. Eur. J. Clin. Microbiol. Infect. Dis..

[5] Qiao W., Wang L., Luo Y., Yang T. (2025). Synthetic approaches and therapeutic applications of FDA-approved antibacterial agents: a comprehensive review from 2003 to 2023. Eur. J. Med. Chem..

[6] Rusu A., Buta E.L. (2021). The development of third-generation tetracycline antibiotics and new perspectives. Pharmaceutics.

[7] Wang Z., Li H. (2024). The tigecycline resistance mechanisms in Gram-negative bacilli. Front. Cell Infect. Microbiol..

[8] Feng J., Zheng Y., Ma W., Weng D., Peng D., Xu Y., Wang Z., Wang X. (2024). A synthetic antibiotic class with a deeply-optimized design for overcoming bacterial resistance. Nat. Commun..

[9] Williford E.E., Xue Y.P., Tang W.K., Li R., Jones K.V., Blake K.S., Blaine H.C., Lian X., Stallings C.L., Tolia N.H., Dantas G., Wencewicz T.A. (2025). C10-benzoate esters of anhydrotetracycline inhibit tetracycline destructases and recover tetracycline antibacterial activity. ACS Infect. Dis..

[10] Furlong-Silva J., Cross S.D., Marriott A.E., Pionnier N., Archer J., Steven A., Merker S.S., Mack M., Hong Y.K., Taylor M.J., Turner J.D. (2021). Tetracyclines improve experimental lymphatic filariasis pathology by disrupting interleukin-4 receptor-mediated lymphangiogenesis. J. Clin. Investig..

[11] Singh B., Ghosh N., Saha D., Sarkar S., Bhattacharyya P., Chaudhury K. (2019). Effect of doxycyline in chronic obstructive pulmonary disease - an exploratory study. Pulm. Pharmacol. Ther..

[12] Huang J., Xue J., Gu J. (2021). EffectS of Minocycline Combined with Tinidazole for Treatment of Chronic Periodontitis. Clin. Investig. Med..

[13] Del Rosso J.Q., Webster G., Weiss J.S., Bhatia N.D., Gold L.S., Kircik L. (2021). Nonantibiotic properties of tetracyclines in rosacea and their clinical implications. J. Clin. Aesthet. Dermatol..

[14] Peukert K., Fox M., Schulz S., Feuerborn C., Frede S., Putensen C., Wrigge H., Kümmerer B.M., David S., Seeliger B., Welte T., Latz E., Klinman D., Wilhelm C., Steinhagen F., Bode C. (2021). Inhibition of caspase-1 with tetracycline ameliorates acute lung injury. Am. J. Respir. Crit. Care Med..

[15] Peukert K., Steinhagen F., Fox M., Feuerborn C., Schulz S., Seeliger B., Schuss P., Schneider M., Frede S., Sauer A., Putensen C., Latz E., Wilhelm C., Bode C. (2022). Tetracycline ameliorates silica-induced pulmonary inflammation and fibrosis via inhibition of caspase-1. Respir. Res..

[16] Garrido-Mesa J., Adams K., Galvez J., Garrido-Mesa N. (2022). Repurposing tetracyclines for acute respiratory distress syndrome (ARDS) and severe COVID-19: a critical discussion of recent publications. Expert Opin. Investig. Drugs.

[17] Enyeji A.M., Arora A., Mangat H.S. (2024). Effective treatment of COVID-19 infection with repurposed drugs: case reports. Viral Immunol..

[18] Bustos-Hamdan A., Bracho-Gallardo J.I., Hamdan-Partida A., Bustos-Martínez J. (2024). Repositioning of antibiotics in the treatment of viral infections. Curr. Microbiol..

[19] Nieman G.F., Zerler B.R. (2001). A role for the anti-inflammatory properties of tetracyclines in the prevention of acute lung injury. Curr. Med. Chem..

[20] Toussirot E., Despaux J., Wendling D. (1997). Do minocycline and other tetracyclines have a place in rheumatology?. Rev. Rhum. Engl. Ed..

[21] Franceschin L., Guidotti A., Mazzetto R., Tartaglia J., Ciolfi C., Alaibac M., Sernicola A. (2024). Repurposing historic drugs for neutrophil-mediated inflammation in skin disorders. Biomolecules.

[22] Radić M., Belančić A., Đogaš H., Vučković M., Gelemanović A., Faour A., Vlak I., Radić J. (2025). Tetracyclines in RHeumatoid Arthritis: Dual Anti-inflammatory and Immunomodulatory Roles, Effectiveness, and Safety insights. Antibiotics.

[23] Singh S., Khanna D., Kalra S. (2021). Minocycline and doxycycline: more than antibiotics. Curr. Mol. Pharmacol..

[24] Santa-Cecília F.V., Leite C.A., Del-Bel E., Raisman-Vozari R. (2019). The neuroprotective effect of doxycycline on neurodegenerative diseases. Neurotox. Res..

[25] Markulin I., Matasin M., Turk V.E., Salković-Petrisic M. (2022). Challenges of repurposing tetracyclines for the treatment of Alzheimer's and Parkinson's disease. J. Neural Transm..

[26] Kumari S., Dhapola R., Reddy D.H. (2023). Apoptosis in Alzheimer's disease: insight into the signaling pathways and therapeutic avenues. Apoptosis.

[27] Conceição M., Di Filippo L.D., Duarte J.L., Beserra F.P., Gremião M.P.D., Chorilli M. (2024). Repurposing doxycycline for Alzheimer's treatment: challenges from a nano-based drug delivery perspective. Brain Behav. Immun. Health.

[28] Hosseini M., Bardaghi Z., Askarpour H., Rajabian A., Mahmoudabady M., Shabab S., Samadi-Noshahr Z., Salmani H. (2024). Minocycline mitigates sepsis-induced neuroinflammation and promotes recovery in male mice: insights into neuroprotection and inflammatory modulation. Physiol. Rep..

[29] Wehrli J.M., Xia Y., Meister L., Tursunova S., Kleim B., Bach D.R., Quednow B.B. (2024). Forget me not: the effect of doxycycline on human declarative memory. Eur. Neuropsychopharmacol..

[30] Finlay A.C., Hobby G.L., SY P.'an, Regna P.P., Routien J.B., Seeley D.B., Shull G.M., Sobin B.A., Solomons I.A., Vinson J.W., Kane J.K. (1950). Terramycin, a new antibiotic. Science.

[31] Stephens C.R., Conover L.H., Hochstein F.A., Regna P.P., Pilgrim F.J., Brunings K.J., Woodward R.B. (1952). J. Am. Chem. Soc..

[32] Donohue J., Dunitz J.D., Trueblood K.N., Webster M.S. (1963). The crystal structure of aureomycin (Chlortetracycline) hydrochloride. Configuration, bond distances and conformation. J. Am. Chem. Soc..

[33] von Wittenau S.M., Blackwood R.K., Conover L.H., Glauert R.H., Woodward R.B. (1965). The stereochemistry at C-5 in oxytetracycline. J. Am. Chem. Soc..

[34] Aleksandrov A., Proft J., Hinrichs W., Simonson T. (2007). Protonation patterns in tetracycline: tet repressor recognition: simulations and experiments. ChemBioChem.

[35] Volkers G., Petruschka L., Hinrichs W. (2011). Recognition of drug degradation products by target proteins: isotetracycline binding to Tet repressor. J. Med. Chem..

[36] Palm G.J., Buchholz I., Werten S., Girbardt B., Berndt L., Delcea M., Hinrichs W. (2020). Thermodynamics, cooperativity and stability of the tetracycline repressor (TetR) upon tetracycline binding. Biochim Biophys. Acta Proteins Proteom..

[37] Barrenechea V., Vargas-Reyes M., Quiliano M., Milón P. (2021). A complementary mechanism of bacterial mRNA translation inhibition by tetracyclines. Front. Microbiol..

[38] Li X., Wang M., Denk T., Buschauer R., Li Y., Beckmann R., Cheng J. (2024). Structural basis for differential inhibition of eukaryotic ribosomes by tigecycline. Nat. Commun..

[39] LaPlante K.L., Dhand A., Wright K., Lauterio M. (2022). Re-establishing the utility of tetracycline-class antibiotics for current challenges with antibiotic resistance. Ann. Med..

[40] Stephens C.R., Murai K., Rennhard H.H., Conover L.H., Brunings K.J. (1958). Hydrogenolysis studies in the tetracycline series—6-deoxytetracyclines. Am. Chem. Soc..

[41] Stephens C.R., Beereboom J.J., Rennhard H.H., Gordon P.N., Murai K., Blackwood R.K., von Wittenau M.S. (1963). 6-Deoxytetracyclines. IV. Preparation, C-6 stereochemistry, and reactions. Am. Chem. Soc..

[42] Blackwood R.K., Beereboom J.J., Rennhard H.H., von Wittenau M.S., Stephens C.R. (1963). J. Am. Chem. Soc..

[43] Church R.F., Schaub R.E., Weiss M.J. (1971). Synthesis of 7-dimethylamino-6-demethyl-6-deoxytetracycline (minocycline) via 9-nitro-6-demethyl-6-deoxytetracycline. J. Org. Chem..

[44] Doan T.L., Fung H.B., Mehta D., Riska P.F. (2006). Tigecycline: a glycylcycline antimicrobial agent. Clin. Ther..

[45] Greer N.D. (2006). Tigecycline (Tygacil): the first in the glycylcycline class of antibiotics. Proc. (Bayl. Univ. Med Cent..

[46] Remash A., Rao P., Shenoy S., Baliga S., Kassim S. (2024). Evaluation of role of Tigecycline among clinically significant multidrug resistant pathogens from a tertiary care hospital. F1000Res.

[47] Wu R., Cui X., Pan R., Li N., Zhang Y., Shu J., Liu Y. (2025). Pathogenic characterization and drug resistance of neonatal sepsis in China: a systematic review and meta-analysis. Eur. J. Clin. Microbiol. Infect. Dis..

[48] Su W., Wang W., Li L., Zhang M., Xu H., Fu C., Pang X., Wang M. (2024). Mechanisms of tigecycline resistance in Gram-negative bacteria: a narrative review. Eng. Microbiol..

[49] Krawczyk S.J., Leśniczak-Staszak M., Gowin E., Szaflarski W. (2024). Mechanistic insights into clinically relevant ribosome-targeting antibiotics. Biomolecules.

[50] Rezaei A., Moqadami A., Khalaj-Kondori M., Feizi M.A.H. (2024). Minocycline induced apoptosis and suppressed expression of matrix metalloproteinases 2 and 9 in the breast cancer MCF-7 cells. Mol. Biol. Rep..

[51] Li J., Qin Y., Zhao C., Zhang Z., Zhou Z. (2023). Tetracycline antibiotics: potential anticancer drugs. Eur. J. Pharmacol..

[52] Mathur S., Srivastava P., Srivastava A., Rai N.K., Abbas S., Kumar A., Tiwari M., Sharma L.K. (2024). Regulation of metastatic potential by drug repurposing and mitochondrial targeting in colorectal cancer cells. BMC Cancer.

[53] Afshari A.R., Mollazadeh H., Sahebkar A. (2020). Minocycline in treating glioblastoma multiforme: far beyond a conventional antibiotic. J. Oncol..

[54] Arriaga M.A., Amieva J.A., Jr, Quintanilla J., Jimenez A., Ledezma J., Lopez S., Martirosyan K.S., Chew S.A. (2023). The application of electrosprayed minocycline-loaded PLGA microparticles for the treatment of glioblastoma. Biotechnol. Bioeng..

[55] Rezaei A., Moqadami A., Khalaj-Kondori M. (2024). Minocycline as a prospective therapeutic agent for cancer and non-cancer diseases: a scoping review. Naunyn Schmiede Arch. Pharmacol..

[56] Iglesias L.P., Soares N., Asth L., Moreira F.A., Aguiar D.C. (2024). Minocycline as a potential anxiolytic drug: systematic review and meta-analysis of evidence in murine models. Behav. Pharmacol..

[57] Cai D.B., Zheng W., Zhang Q.E., Ng C.H., Ungvari G.S., Huang X., Xiang Y.T. (2020). Minocycline for depressive symptoms: a meta-analysis of randomized, double-blinded, placebo-controlled trials. Psychiatr. Q.

[58] Panizzutti B., Skvarc D., Lin S., Croce S., Meehan A., Bortolasci C.C., Marx W., Walker A.J., Hasebe K., Kavanagh B.E., Morris M.J., Mohebbi M., Turner A., Gray L., Berk L., Walder K., Berk M., Dean O.M. (2023). Minocycline as treatment for psychiatric and neurological conditions: a systematic review and meta-analysis. Int. J. Mol. Sci..

[59] Lanzenberger R., Kraus C. (2024). Relative effectiveness of antidepressant treatments in treatment-resistant depression: a systematic review and network meta-analysis of randomized controlled trials. Neuropsychopharmacology.

[60] Hunter C.L., Quintero E.M., Gilstrap L., Bhat N.R., Granholm A.C. (2004). Minocycline protects basal forebrain cholinergic neurons from mu p75-saporin immunotoxic lesioning. Eur. J. Neurosci..

[61] Choi Y., Kim H.S., Shin K.Y., Kim E.M., Kim M., Kim H.S., Park C.H., Jeong Y.H., Yoo J., Lee J.P., Chang K.A., Kim S., Suh Y.H. (2007). Minocycline attenuates neuronal cell death and improves cognitive impairment in Alzheimer's disease models. Neuropsychopharmacology.

[62] Seabrook T.J., Jiang L., Maier M., Lemere C.A. (2006). Minocycline affects microglia activation, Abeta deposition, and behavior in APP-tg mice. Glia.

[63] Fan R., Xu F., Previti M.L., Davis J., Grande A.M., Robinson J.K., Van Nostrand W.E. (2007). Minocycline reduces microglial activation and improves behavioral deficits in a transgenic model of cerebral microvascular amyloid. J. Neurosci..

[64] Noble W., Garwood C., Stephenson J., Kinsey A.M., Hanger D.P., Anderton B.H. (2009). Minocycline reduces the development of abnormal tau species in models of Alzheimer's disease. FASEB J..

[65] Howard R., Zubko O., Bradley R., Harper E., Pank L., O'Brien J., Fox C., Tabet N., Livingston G., Bentham P., McShane R., Burns A., Ritchie C., Reeves S., Lovestone S., Ballard C., Noble W., Nilforooshan R., Wilcock G., Gray R. (2020). Minocycline in Alzheimer disease efficacy (MADE) Trialist Group. Minocycline at 2 different dosages vs placebo for patients with mild alzheimer disease: a randomized clinical trial. JAMA Neurol..

[66] Abdo Qaid E.Y., Abdullah Z., Zakaria R., Long I. (2024). Minocycline mitigates tau pathology via modulating the TLR-4/NF-кβ signalling pathway in the hippocampus of neuroinflammation rat model. Neurol. Res..

[67] Vicente M.C., Paneghini J.L., Stabile A.M., Amorim M., Anibal Silva C.E., Patrone L.G.A., Cunha T.M., Bícego K.C., Almeida M.C., Carrettiero D.C., Gargaglioni L.H. (2023). Inhibition of pro-inflammatory microglia with minocycline improves cognitive and sleep-wake dysfunction under respiratory stress in a sporadic model for Alzheimer's disease. J. Alzheimers Dis..

[68] Gholami Mahmoudian Z., Komaki A., Rashidi I., Amiri I., Ghanbari A. (2022). The effect of minocycline on beta-amyloid-induced memory and learning deficit in male rats: a behavioral, biochemical, and histological study. J. Chem. Neuroanat..

[69] Amirahmadi S., Farimani F.D., Akbarian M., Mirzavi F., Eshaghi Ghalibaf M.H., Rajabian A., Hosseini M. (2022). Minocycline attenuates cholinergic dysfunction and neuro-inflammation-mediated cognitive impairment in scopolamine-induced Alzheimer's rat model. Inflammopharmacology.

[70] Gholami Mahmoudian Z., Ghanbari A., Rashidi I., Amiri I., Komaki A. (2023). Minocycline effects on memory and learning impairment in the beta-amyloid-induced Alzheimer's disease model in male rats using behavioral, biochemical, and histological methods. Eur. J. Pharmacol..

[71] Kater M.S.J., Huffels C.F.M., Oshima T., Renckens N.S., Middeldorp J., Boddeke E.W.G.M., Smit A.B., Eggen B.J.L., Hol E.M., Verheijen M.H.G. (2023). Prevention of microgliosis halts early memory loss in a mouse model of Alzheimer's disease. Brain Behav. Immun..

[72] Balducci C., Mehdawy B., Mare L., Giuliani A., Lorenzini L., Sivilia S., Giardino L., Calzà L., Lanzillotta A., Sarnico I., Pizzi M., Usiello A., Viscomi A.R., Ottonello S., Villetti G., Imbimbo B.P., Nisticò G., Forloni G., Nisticò R. (2011). The γ-secretase modulator CHF5074 restores memory and hippocampal synaptic plasticity in plaque-free Tg2576 mice. J. Alzheimers Dis..

[73] Giraldo-Berrio D., Jimenez-Del-Rio M., Velez-Pardo C. (2024). Minocycline mitigates Aβ and TAU pathology, neuronal dysfunction, and death in the PSEN1 E280A cholinergic-like neurons model of familial Alzheimer's disease. Neuropharmacology.

[74] Chan S.Y., Capitão L., Probert F., Klinge C., Hoeckner S., Harmer C.J., Cowen P.J., Anthony D.C., Burnet P.W.J. (2020). A single administration of the antibiotic, minocycline, reduces fear processing and improves implicit learning in healthy volunteers: analysis of the serum metabolome. Transl. Psychiatry.

[75] Homsi S., Piaggio T., Croci N., Noble F., Plotkine M., Marchand-Leroux C., Jafarian-Tehrani M. (2010). Blockade of acute microglial activation by minocycline promotes neuroprotection and reduces locomotor hyperactivity after closed head injury in mice: a twelve-week follow-up study. J. Neurotrauma.

[76] Whitney K., Nikulina E., Rahman S.N., Alexis A., Bergold P.J. (2021). Delayed dosing of minocycline plus N-acetylcysteine reduces neurodegeneration in distal brain regions and restores spatial memory after experimental traumatic brain injury. Exp. Neurol..

[77] Lu Q., Xiong J., Yuan Y., Ruan Z., Zhang Y., Chai B., Li L., Cai S., Xiao J., Wu Y., Huang P., Zhang H. (2022). Minocycline improves the functional recovery after traumatic brain injury via inhibition of aquaporin-4. Int. J. Biol. Sci..

[78] Scott G., Zetterberg H., Jolly A., Cole J.H., De Simoni S., Jenkins P.O., Feeney C., Owen D.R., Lingford-Hughes A., Howes O., Patel M.C., Goldstone A.P., Gunn R.N., Blennow K., Matthews P.M., Sharp D.J. (2018). Minocycline reduces chronic microglial activation after brain trauma but increases neurodegeneration. Brain.

[79] Koulaeinejad N., Haddadi K., Ehteshami S., Shafizad M., Salehifar E., Emadian O., Ali Mohammadpour R., Ala S. (2019). Effects of minocycline on neurological outcomes in patients with acute traumatic brain injury: a pilot study. Iran. J. Pharm. Res..

[80] Bergold P.J., Furhang R., Lawless S. (2023). Treating traumatic brain injury with minocycline. Neurotherapeutics.

[81] Ritter K., Somnuke P., Hu L., Griemert E.V., Schäfer M.K.E. (2024). Current state of neuroprotective therapy using antibiotics in human traumatic brain injury and animal models. BMC Neurosci..

[82] Lee S.M., Yune T.Y., Kim S.J., Park D.W., Lee Y.K., Kim Y.C., Oh Y.J., Markelonis G.J., Oh T.H. (2003). Minocycline reduces cell death and improves functional recovery after traumatic spinal cord injury in the rat. J. Neurotrauma.

[83] Hurlbert Wells J.E., Fehlings R.J., Yong M.G. (2003). VW. Neuroprotection by minocycline facilitates significant recovery from spinal cord injury in mice. Brain.

[84] Papa S., Caron I., Erba E., Panini N., De Paola M., Mariani A., Colombo C., Ferrari R., Pozzer D., Zanier E.R., Pischiutta F., Lucchetti J., Bassi A., Valentini G., Simonutti G., Rossi F., Moscatelli D., Forloni G., Veglianese P. (2016). Early modulation of pro-inflammatory microglia by minocycline loaded nanoparticles confers long lasting protection after spinal cord injury. Biomaterials.

[85] Papa S., Rossi F., Ferrari R., Mariani A., De Paola M., Caron I., Fiordaliso F., Bisighini C., Sammali E., Colombo C., Gobbi M., Canovi M., Lucchetti J., Peviani M., Morbidelli M., Forloni G., Perale G., Moscatelli D., Veglianese P. (2013). Selective nanovector mediated treatment of activated proinflammatory microglia/macrophages in spinal cord injury. ACS Nano.

[86] Gu J., Cai X., Raza F., Zafar H., Chu B., Yuan H., Wang T., Wang J., Feng X. (2024). Preparation of a minocycline polymer micelle thermosensitive gel and its application in spinal cord injury. Nanoscale Adv..

[87] Casha S., Zygun D., McGowan M.D., Bains I., Yong V.W., Hurlbert R.J. (2012). Results of a phase II placebo-controlled randomized trial of minocycline in acute spinal cord injury. Brain.

[88] Casha S., Rice T., Stirling D.P., Silva C., Gnanapavan S., Giovannoni G., Hurlbert R.J., Yong V.W. (2018). Cerebrospinal fluid biomarkers in human spinal cord injury from a phase II minocycline trial. J. Neurotrauma.

[89] Sanchez Mejia R.O., Ona V.O., Li M., Friedlander R.M. (2001). Minocycline reduces traumatic brain injury-mediated caspase-1 activation, tissue damage, and neurological dysfunction. Neurosurgery.

[90] Joaquim A.F., Daniel J.W., Schroeder G.D., Vaccaro A.R. (2020). Neuroprotective agents as an adjuvant treatment in patients with acute spinal cord injuries: a qualitative systematic review of randomized trials. Clin. Spine Surg..

[91] Hu C.W., Li Z.Y., Zhu K., Dai Y.X., Zhang C., Sun Y.L., Shi Q., Cui X.J., Yao M. (2025). Exploring the effectiveness and potential pharmacological mechanism of minocycline for spinal cord injury through meta-analysis and network pharmacology. Curr. Neuropharmacol..

[92] Sherrod B.A., Porche K., Condie C.K., Dailey A.T. (2024). Pharmacologic therapy for spinal cord injury. Clin. Spine Surg..

[93] Atigari O.V., Hogan C., Healy D. (2013). Doxycycline and suicidality. BMJ Case Rep..

[94] Ben-Azu B., Omogbiya I.A., Aderibigbe A.O., Umukoro S., Ajayi A.M., Iwalewa E.O. (2018). Doxycycline prevents and reverses schizophrenic-like behaviors induced by ketamine in mice via modulation of oxidative, nitrergic and cholinergic pathways. Brain Res. Bull..

[95] Sales A.J., Joca S.R.L., Del Bel E., Guimarães F.S. (2024). The antidepressant-like effect of doxycycline is associated with decreased nitric oxide metabolite levels in the prefrontal cortex. Behav. Brain Res..

[96] Chaves Filho A.J.M., Cunha N.L., Rodrigues P.A., de Souza A.G., Soares M.V., Jucá P.M., de Queiroz T., Clemente D.C.D.S., Mottin M., Andrade C.H., Peixoto C.A., Macedo D.S. (2021). Doxycycline reverses cognitive impairment, neuroinflammation and oxidative imbalance induced by D-amphetamine mania model in mice: a promising drug repurposing for bipolar disorder treatment?. Eur. Neuropsychopharmacol..

[97] Tagliavini F., Forloni G., Colombo L., Rossi G., Girola L., Canciani B., Angeretti N., Giampaolo L., Peressini E., Awan T., De Gioia L., Ragg E., Bugiani O., Salmona M. (2000). Tetracycline affects abnormal properties of synthetic PrP peptides and PrP(Sc) in vitro. J. Mol. Biol..

[98] Forloni G., Colombo L., Girola L., Tagliavini F., Salmona M. (2001). Anti-amyloidogenic activity of tetracyclines: studies in vitro. FEBS Lett..

[99] Stoilova T., Colombo L., Forloni G., Tagliavini F., Salmona M. (2013). A new face for old antibiotics: tetracyclines in treatment of amyloidoses. J. Med Chem..

[100] Gautieri A., Beeg M., Gobbi M., Rigoldi F., Colombo L., Salmona M. (2019). The anti-amyloidogenic action of doxycycline: a molecular dynamics study on the interaction with Aβ42. Int J. Mol. Sci..

[101] Forloni G., La Vitola P., Balducci C. (2022). *Oligomeropathies*, inflammation and prion protein binding. Front. Neurosci..

[102] Jarrett J.T., Lansbury P.T., Jr (1993). Seeding "one-dimensional crystallization" of amyloid: a pathogenic mechanism in Alzheimer's disease and scrapie?. Cell.

[103] Come J.H., Fraser P.E., Lansbury P.T. (1993). A kinetic model for amyloid formation in the prion diseases: importance of seeding. Proc. Natl. Acad. Sci. USA.

[104] Forloni G., Artuso V., La Vitola P., Balducci C. (2016). Oligomeropathies and pathogenesis of Alzheimer and Parkinson’s diseases. Mov. Disord..

[105] Medina L., González-Lizárraga F., Dominguez-Meijide A., Ploper D., Parrales V., Sequeira S., Cima-Omori M.S., Zweckstetter M., Del Bel E., Michel P.P., Outeiro T.F., Raisman-Vozari R., Chehín R., Socias S.B. (2021). Doxycycline interferes with Tau aggregation and reduces its neuronal toxicity. Front. Aging Neurosci..

[106] González-Lizárraga F., Socías S.B., Ávila C.L., Torres-Bugeau C.M., Barbosa L.R., Binolfi A., Sepúlveda-Díaz J.E., Del-Bel E., Fernandez C.O., Papy-Garcia D., Itri R., Raisman-Vozari R., Chehín R.N. (2017). Repurposing doxycycline for synucleinopathies: remodelling of α-synuclein oligomers towards non-toxic parallel beta-sheet structured species. Sci. Rep..

[107] Rose C., Tomas-Grau R.H., Zabala B., Michel P.P., Brunel J.M., Chehín R., Raisman-Vozari R., Ferrié L., Figadère B. (2024). C9-FUnctionalized Doxycycline Analogs as Drug Candidates to Prevent Pathological α-Synuclein Aggregation and Neuroinflammation in Parkinson's disease degeneration. ChemMedChem.

[108] Balducci C., Santamaria G., La Vitola P., Brandi E., Grandi F., Viscomi A.R., Beeg M., Gobbi M., Salmona M., Ottonello S., Forloni G. (2018). Doxycycline counteracts neuroinflammation restoring memory in Alzheimer's disease mouse models. Neurobiol. Aging.

[109] Balducci C., Forloni G. (2019). Doxycycline for Alzheimer's disease: fighting β-amyloid oligomers and neuroinflammation. Front. Pharmacol..

[110] Zhang G.B., Feng Y.H., Wang P.Q., Song J.H., Wang P., Wang S.A. (2015). A study on the protective role of doxycycline upon dopaminergic neuron of LPS-PD rat model rat. Eur. Rev. Med Pharmacol. Sci..

[111] Wiggins-Dohlvik K., Stagg H.W., Han M.S., Alluri H., Oakley R.P., Anasooya Shaji C., Davis M.L., Tharakan B. (2016). Doxycycline attenuates lipopolysaccharide-induced microvascular endothelial cell derangements. Shock.

[112] Mello B.S.F., Chaves Filho A.J.M., Custódio C.S., Rodrigues P.A., Carletti J.V., Vasconcelos S.M.M., Sousa F.C.F., Sanders L.L.O., Macedo D.S. (2021). Doxycycline at subantimicrobial dose combined with escitalopram reverses depressive-like behavior and neuroinflammatory hippocampal alterations in the lipopolysaccharide model of depression. J. Affect Disord..

[113] Gomez-Murcia V., Carvalho K., Thiroux B., Caillierez R., Besegher M., Sergeant N., Buée L., Faivre E., Blum D. (2022). Impact of chronic doxycycline treatment in the APP/PS1 mouse model of Alzheimer's disease. Neuropharmacology.

[114] Morsy S.A.A., Fathelbab M.H., El-Sayed N.S., El-Habashy S.E., Aly R.G., Harby S.A. (2024). Doxycycline-loaded calcium phosphate nanoparticles with a pectin coat can ameliorate lipopolysaccharide-induced neuroinflammation via enhancing AMPK. J. Neuroimmune Pharmacol..

[115] Loeb M.B., Molloy D.W., Smieja M., Standish T., Goldsmith C.H., Mahony J., Smith S., Borrie M., Decoteau E., Davidson W., McDougall A., Gnarpe J., O'DONNell M., Chernesky M. (2004). A randomized, controlled trial of doxycycline and rifampin for patients with Alzheimer's disease. J. Am. Geriatr. Soc..

[116] Molloy D.W., Standish T.I., Zhou Q., Guyatt G. (2013). DARAD Study Group. A multicenter, blinded, randomized, factorial controlled trial of doxycycline and rifampin for treatment of Alzheimer's disease: the DARAD trial. Int. J. Geriatr. Psychiatry.

[117] Gianni L., Bellotti V., Gianni A.M., Merlini G. (1995). New drug therapy of amyloidoses: resorption of AL-type deposits with 4′-iodo-4′-deoxydoxorubicin. Blood.

[118] Tagliavini F., Forloni G., Colombo L., Rossi G., Girola L., Canciani B., Angeretti N., Giampaolo L., Peressini E., Awan T., De Gioia L., Ragg E., Bugiani O., Salmona M. (2000). Tetracycline affects abnormal properties of synthetic PrP peptides and PrP(Sc) in vitro. J. Mol. Biol..

[119] Forloni G., Iussich S., Awan T., Colombo L., Angeretti N., Girola L., Bertani I., Poli G., Caramelli M., Bruzzone M.G., Farina L., Limido L., Rossi G., Giaccone G., Ironside J.W., Bugiani O., Salmona M., Tagliavini F. (2002). Tetracyclines affect prion infectivity. Proc. Natl. Acad. Sci. USA.

[120] De Luigi A., Colombo L., Diomede L., Capobianco R., Mangieri M., Miccolo C., Limido L., Forloni G., Tagliavini F., Salmona M. (2008). The efficacy of tetracyclines in peripheral and intracerebral prion infection. PLOS One.

[121] Tagliavini F., Prion therapy: Tetracyclic compounds in animal models and patients with Creutzfeldt-Jakob disease 4, Issue 4S_Part_5.

[122] Varges D., Manthey H., Heinemann U., Ponto C., Schmitz M., Schulz-Schaeffer W.J., Krasnianski A., Breithaupt M., Fincke F., Kramer K., Friede T., Zerr I. (2017). Doxycycline in early CJD: a double-blinded randomised phase II and observational study. J. Neurol. Neurosurg. Psychiatry.

[123] Haïk S., Marcon G., Mallet A., Tettamanti M., Welaratne A., Giaccone G., Azimi S., Pietrini V., Fabreguettes J.R., Imperiale D., Cesaro P., Buffa C., Aucan C., Lucca U., Peckeu L., Suardi S., Tranchant C., Zerr I., Houillier C., Redaelli V., Vespignani H., Campanella A., Sellal F., Krasnianski A., Seilhean D., Heinemann U., Sedel F., Canovi M., Gobbi M., Di Fede G., Laplanche J.L., Pocchiari M., Salmona M., Forloni G., Brandel J.P., Tagliavini F. (2014). Doxycycline in Creutzfeldt-Jakob disease: a phase 2, randomised, double-blind, placebo-controlled trial. Lancet Neurol..

[124] Forloni G., Tettamanti M., Lucca U., Albanese Y., Quaglio E., Chiesa R., Erbetta A., Villani F., Redaelli V., Tagliavini F., Artuso V., Roiter I. (2015). Preventive study in subjects at risk of fatal familial insomnia: Innovative approach to rare diseases. Prion.

[125] Forloni G., Roiter I., Artuso V., Marcon M., Colesso W., Luban E., Lucca U., Tettamanti M., Pupillo E., Redaelli V., Mariuzzo F., Boscolo Buleghin G., Mariuzzo A., Tagliavini F., Chiesa R., Ambrosini A. (2022). Preventive pharmacological treatment in subjects at risk for fatal familial insomnia: science and public engagement. Prion.

[126] Zerr I., Ladogana A., Mead S., Hermann P., Forloni G., Appleby B.S. (2024). Creutzfeldt-Jakob disease and other prion diseases. Nat. Rev. Dis. Prim..

[127] Forloni G., Balducci C. (2018). Alzheimer's disease, oligomers, and inflammation. J. Alzheimers Dis..

[128] Zhang W., Xiao D., Mao Q., Xia H. (2023). Role of neuroinflammation in neurodegeneration development. Signal Transduct. Target Ther..

[129] Forloni G. (2023). Alpha synuclein: neurodegeneration and inflammation. Int. J. Mol. Sci..

[130] Vida H., Sahar M., Nikdouz A., Arezoo H. (2024). Chemokines in neurodegenerative diseases. Immunol. Cell Biol..

[131] Chamani S., Bianconi V., Tasbandi A., Pirro M., Barreto G.E., Jamialahmadi T., Sahebkar A. (2020). Resolution of inflammation in neurodegenerative diseases: the role of resolvins. Mediat. Inflamm..

[132] Soto C., Pritzkow S. (2018). Protein misfolding, aggregation, and conformational strains in neurodegenerative diseases. Nat. Neurosci..

[133] Cho Y., Son H.J., Kim E.M., Choi J.H., Kim S.T., Ji I.J., Choi D.H., Joh T.H., Kim Y.S., Hwang O. (2009). Doxycycline is neuroprotective against nigral dopaminergic degeneration by a dual mechanism involving MMP-3. Neurotox. Res..

[134] Lazzarini M., Martin S., Mitkovski M., Vozari R.R., Stühmer W., Bel E.D. (2013). Doxycycline restrains glia and confers neuroprotection in a 6-OHDA Parkinson model. Glia.

[135] Zhang G.B., Feng Y.H., Wang P.Q., Song J.H., Wang P., Wang S.A. (2015). A study on the protective role of doxycycline upon dopaminergic neuron of LPS-PD rat model rat. Eur. Rev. Med. Pharmacol. Sci..

[136] Dos Santos Pereira M., do, Nascimento G.C., Bortolanza M., Michel P.P., Raisman-Vozari R., Del Bel E. (2022). Doxycycline attenuates l-DOPA-induced dyskinesia through an anti-inflammatory effect in a hemiparkinsonian mouse model. Front. Pharmacol..

[137] Forloni G., La Vitola P., Cerovic M., Balducci C. (2021). Inflammation and Parkinson's disease pathogenesis: mechanisms and therapeutic insight. Prog. Mol. Biol. Transl. Sci..

[138] La Vitola P., Artioli L., Cerovic M., Poletto C., Dacomo L., Leva S., Balducci C., Forloni G. (2023). Repositioning doxycycline for treating synucleinopathies: evidence from a pre-clinical mouse model. Park. Relat. Disord..

[139] Sims J.R., Zimmer J.A., Evans C.D., Lu M., Ardayfio P., Sparks J., Wessels A.M., Shcherbinin S., Wang H., Monkul Nery E.S., Collins E.C., Solomon P., Salloway S., Apostolova L.G., Hansson O., Ritchie C., Brooks D.A., Mintun M., Skovronsky D.M., TRAILBLAZER-ALZ 2 Investigators (2023). Donanemab in early symptomatic Alzheimer disease: the TRAILBLAZER-ALZ 2 randomized clinical trial. JAMA.

[140] van Dyck C.H., Swanson C.J., Aisen P., Bateman R.J., Chen C., Gee M., Kanekiyo M., Li D., Reyderman L., Cohen S., Froelich L., Katayama S., Sabbagh M., Vellas B., Watson D., Dhadda S., Irizarry M., Kramer L.D., Iwatsubo T. (2023). Lecanemab in early alzheimer's disease. N. Engl. J. Med..

[141] Schenk D., Barbour R., Dunn W., Gordon G., Grajeda H., Guido T., Hu K., Huang J., Johnson-Wood K., Khan K., Kholodenko D., Lee M., Liao Z., Lieberburg I., Motter R., Mutter L., Soriano F., Shopp G., Vasquez N., Vandevert C., Walker S., Wogulis M., Yednock T., Games D., Seubert P. (1999). Immunization with amyloid-beta attenuates Alzheimer-disease-like pathology in the PDAPP mouse. Nature.

[142] Zhang Y., Chen J., Li Y., Jiao B., Luo S. (2024). Disease-modifying therapies for Alzheimer's disease: Clinical trial progress and opportunity. Ageing Res. Rev..

[143] Sato S., Hatakeyama N., Fujikoshi S., Katayama S., Katagiri H., Sims J.R. (2024). Donanemab in Japanese patients with early alzheimer's disease: subpopulation analysis of the TRAILBLAZER-ALZ 2 randomized trial. Neurol. Ther..

[144] Jin M., Noble J.M. (2024). What's in It for Me? Contextualizing the potential clinical impacts of lecanemab, donanemab, and other anti-β-amyloid monoclonal antibodies in early Alzheimer's disease. eNeuro.

[145] Jicha G.A., Abner E.L., Coskun E.P., Huffmyer M.J., Tucker T.C., Nelson P.T. (2024). Perspectives on the clinical use of anti-amyloid therapy for the treatment of Alzheimer's disease: Insights from the fields of cancer, rheumatology, and neurology. Alzheimers Dement..

[146] Ramanan V.K., Armstrong M.J., Choudhury P., Coerver K.A., Hamilton R.H., Klein B.C., Wolk D.A., Wessels S.R., Jones L.K., Jr; A.A.N. (2023). Quality Committee. Antiamyloid monoclonal antibody therapy for alzheimer disease: emerging issues in neurology. Neurology.

[147] Couzin-Frankel J. (2024). Alzheimer's drug approvals create prescribing dilemmas. Science.

[148] Shields L.B.E., Hust H., Cooley S.D., Cooper G.E., Hart R.N., Dennis B.C., Freeman S.W., Cain J.F., Shang W.Y., Wasz K.M., Orr A.T., Shields C.B., Barve S.S., Pugh K.G. (2024). Initial Experience with lecanemab and lessons learned in 71 patients in a regional medical center. J. Prev. Alzheimers Dis..

[149] Xing X., Zhang X., Wang K., Wang Z., Feng Y., Li X., Hua Y., Zhang L., Dong X. (2025). Post-marketing safety concerns with lecanemab: a pharmacovigilance study based on the FDA adverse event reporting system database. Alzheimers Res. Ther..

[150] Arroyo-Pacheco N., Sarmiento-Blanco S., Vergara-Cadavid G., Castro-Leones M., Contreras-Puentes N. (2024). Monoclonal therapy with lecanemab in the treatment of mild Alzheimer's disease: a systematic review and meta-analysis. Ageing Res. Rev..

[151] Jack C.R., Jr, Andrews J.S., Beach T.G., Buracchio T., Dunn B., Graf A., Hansson O., Ho C., Jagust W., McDade E., Molinuevo J.L., Okonkwo O.C., Pani L., Rafii M.S., Scheltens P., Siemers E., Snyder H.M., Sperling R., Teunissen C.E., Carrillo M.C. (2024). Revised criteria for diagnosis and staging of Alzheimer's disease: Alzheimer's Association Workgroup. Alzheimers Dement..

[152] Therriault J., Janelidze S., Benedet A.L., Ashton N.J., Arranz Martínez J., Gonzalez-Escalante A., Bellaver B., Alcolea D., Vrillon A., Karim H., Mielke M.M., Hyung Hong C., Roh H.W., Contador J., Puig Pijoan A., Algeciras-Schimnich A., Vemuri P., Graff-Radford J., Lowe V.J., Karikari T.K., Jonaitis E., Brum W., Tissot C., Servaes S., Rahmouni N., Macedo A.C., Stevenson J., Fernandez-Arias J., Wang Y.T., Woo M.S., Friese M.A., Jia W.L., Dumurgier J., Hourregue C., Cognat E., Ferreira P.L., Vitali P., Johnson S., Pascoal T.A., Gauthier S., Lleó A., Paquet C., Petersen R.C., Salmon D., Mattsson-Carlgren N., Palmqvist S., Stomrud E., Galasko D., Son S.J., Zetterberg H., Fortea J., Suárez-Calvet M., Jack C.R., Jr, Blennow K., Hansson O., Rosa-Neto P. (2024). Diagnosis of Alzheimer's disease using plasma biomarkers adjusted to clinical probability. Nat. Aging.

[153] Cummings J., Apostolova L., Rabinovici G.D., Atri A., Aisen P., Greenberg S., Hendrix S., Selkoe D., Weiner M., Petersen R.C., Salloway S. (2023). Lecanemab: appropriate use recommendations. J. Prev. Alzheimers Dis..

[154] Logroscino G., Urso D., Gnoni V., Giugno A., Vilella D., Castri A., Barone R., Nigro S., Zecca C., De Blasi R., Introna A. (2025 Jan). Mild cognitive impairment and early Alzheimer's disease eligibility for disease modification therapies in a tertiary centre for cognitive disorders: a simultaneous real-word study on aducanumab and lecanemab. Eur. J. Neurol..

[155] Forloni G. (2020). Alzheimer's disease: from basic science to precision medicine approach. BMJ Neurol. Open.

[156] Lista S., Imbimbo B.P., Grasso M., Fidilio A., Emanuele E., Minoretti P., López-Ortiz S., Martín-Hernández J., Gabelle A., Caruso G., Malaguti M., Melchiorri D., Santos-Lozano A., Imbimbo C., Heneka M.T., Caraci F. (2024). Tracking neuroinflammatory biomarkers in Alzheimer's disease: a strategy for individualized therapeutic approaches?. J. Neuroinflamm..

[157] Arnold S.E., Hyman B.T., Betensky R.A., Dodge H.H. (2024). Pathways to personalized medicine-Embracing heterogeneity for progress in clinical therapeutics research in Alzheimer's disease. Alzheimers Dement..

[158] Kumar R., Waisberg E., Ong J., Paladugu P., Amiri D., Saintyl J., Yelamanchi J., Nahouraii R., Jagadeesan R., Tavakkoli A. (2024). Artificial intelligence-based methodologies for early diagnostic precision and personalized therapeutic strategies in neuro-ophthalmic and neurodegenerative pathologies. Brain Sci..

[159] Tournier B.B., Sorce S., Marteyn A., Ghidoni R., Benussi L., Binetti G., Herrmann F.R., Krause K.H., Zekry D. (2024). CCR5 deficiency: Decreased neuronal resilience to oxidative stress and increased risk of vascular dementia. Alzheimers Dement..

[160] Li J., Xia D., Cui M., Wang Y., Li J., Jin L., Chen X., Suo C., Jiang Y. (2024). Disease trajectories before dementia: evidence from a large-scale community-based prospective study. Br. J. Psychiatry.

[161] Choi J., Beroncal E.L., Chernega T., Brooks H.J., Kennedy J.L., Fisher C.E., Flint A.J., Herrmann N., Lanctôt K.L., Mah L., Mulsant B.H., Pollock B.G., Rajji T.K., Andreazza A.C. (2024). PACt-MD Study Group. Exploring mitochondrial blood-based and genetic markers in older adults with mild cognitive impairment and remitted major depressive disorder. Transl. Psychiatry.

[162] Du W., Yu S., Liu R., Kong Q., Hao X., Liu Y. (2025). Precision prediction of Alzheimer's disease: integrating mitochondrial energy metabolism and immunological insights. J. Mol. Neurosci..

[163] Bigio B., Lima-Filho R.A.S., Barnhill O., Sudo F.K., Drummond C., Assunção N., Vanderborght B., Beasley J., Young S., Korman A., Jones D.R., Sultzer D.L., Ferreira S.T., Mattos P., Head E., Tovar-Moll F., De Felice F.G., Lourenco M.V., Nasca C. (2025). Sex differences in mitochondrial free-carnitine levels in subjects at-risk and with Alzheimer's disease in two independent study cohorts. Mol. Psychiatry.

[164] Huang Y.L., Tsai T.H., Shen Z.Q., Chan Y.H., Tu C.W., Tung C.Y., Wang P.N., Tsai T.F. (2025). Transcriptomic predictors of rapid progression from mild cognitive impairment to Alzheimer's disease. Alzheimers Res. Ther..

[165] Rahman M.A., Rahman M.D.H., Rhim H., Kim B. (2024). Drug target to alleviate mitochondrial dysfunctions in Alzheimer's disease: recent advances and therapeutic implications. Curr. Neuropharmacol..

[166] Li Vok, Han Y., Kaistha T., Zhang Q., Downey J., Gozes I., Lam J.C.K. (2025). DeepDrug as an expert guided and AI driven drug repurposing methodology for selecting the lead combination of drugs for Alzheimer's disease. Sci. Rep..

[167] Dubois B., Villain N., Frisoni G.B., Rabinovici G.D., Sabbagh M., Cappa S., Bejanin A., Bombois S., Epelbaum S., Teichmann M., Habert M.O., Nordberg A., Blennow K., Galasko D., Stern Y., Rowe C.C., Salloway S., Schneider L.S., Cummings J.L., Feldman H.H. (2021). Clinical diagnosis of Alzheimer's disease: recommendations of the international working group. Lancet Neurol..

[168] Dubois B., Villain N., Schneider L., Fox N., Campbell N., Galasko D., Kivipelto M., Jessen F., Hanseeuw B., Boada M., Barkhof F., Nordberg A., Froelich L., Waldemar G., Frederiksen K.S., Padovani A., Planche V., Rowe C., Bejanin A., Ibanez A., Cappa S., Caramelli P., Nitrini R., Allegri R., Slachevsky A., de Souza L.C., Bozoki A., Widera E., Blennow K., Ritchie C., Agronin M., Lopera F., Delano-Wood L., Bombois S., Levy R., Thambisetty M., Georges J., Jones D.T., Lavretsky H., Schott J., Gatchel J., Swantek S., Newhouse P., Feldman H.H., Frisoni G.B. (2024). Alzheimer disease as a clinical-biological construct-an international working group recommendation. JAMA Neurol..

[169] Ghanbarian E., Khorsand B., Petersen K.K., Nallapu B.T., Sajjadi S.A., Lipton R.B., Ezzati A. (2024). CSF inflammatory cytokines as prognostic indicators for cognitive decline across Alzheimer's disease spectrum. medRxiv.

[170] Charisis S., Mourtzi N., Scott M.R., Ntanasi E., Mamalaki E., Hatzimanolis A., Ramirez A., Lambert J.C., Yannakoulia M., Kosmidis M., Dardiotis E., Hadjigeorgiou G., Sakka P., Satizabal C.L., Beiser A., Yang Q., Georgakis M.Κ, Seshadri S., Scarmeas N. (2025). Genetic predisposition to high circulating levels of interleukin 6 and risk for Alzheimer's disease. Discovery and replication. J. Prev. Alzheimers Dis..

[171] Abbatecola A.M., Giuliani A., Biscetti L., Scisciola L., Battista P., Barbieri M., Sabbatinelli J., Olivieri F. (2024). Circulating biomarkers of inflammaging and Alzheimer's disease to track age-related trajectories of dementia: Can we develop a clinically relevant composite combination?. Ageing Res. Rev..

[172] Robles-Vera I., de la Visitación N., Toral M., Sánchez M., Romero M., Gómez-Guzmán M., Vargas F., Duarte J., Jiménez R. (2021). Changes in gut microbiota induced by doxycycline influence in vascular function and development of hypertension in DOCA-salt rats. Nutrients.

[173] Cacabelos R., Martínez-Iglesias O., Cacabelos N., Carrera I., Corzo L., Naidoo V. (2024). Therapeutic options in alzheimer's disease: from classic acetylcholinesterase inhibitors to multi-target drugs with pleiotropic activity. Life.

[174] Ferretti G., Serafini S., Angiolillo A., Monterosso P., Di Costanzo A., Matrone C. (2023). Advances in peripheral blood biomarkers of patients with Alzheimer's disease: moving closer to personalized therapies. Biomed. Pharmacol..

[175] EMA, 2019, Committee for Medicinal Products for Human Use (CHMP) Guideline on the clinical investigation of medicines for the treatment of Alzheimer’s disease. Available: 〈https://www〉. ema. europa. eu/.

[176] Gauthier S., Alam J., Fillit H., Iwatsubo T., Liu-Seifert H., Sabbagh M., Salloway S., Sampaio C., Sims J.R., Sperling B., Sperling R., Welsh-Bohmer K.A., Touchon J., Vellas B., Aisen P. (2019). Combination therapy for Alzheimer's disease: perspectives of the EU/US CTAD task force. J. Prev. Alzheimers Dis..

[177] Cummings J.L., Osse A.M.L., Kinney J.W., Cammann D., Chen J. (2024). Alzheimer's disease: combination therapies and clinical trials for combination therapy development. CNS Drugs.

[178] Cummings J., Gold M., Mintun M., Irizarry M., von Eschenbach A., Hendrix S., Berry D., Sampaio C., Sink K., Landen J., Kivipelto M., Grundman M., Arnold S.E., Green A., Partrick K., Nisenbaum L., Burstein A., Fillit H. (2025). Key considerations for combination therapy in Alzheimer's clinical trials: perspectives from an expert advisory board convened by the Alzheimer's drug discovery foundation. J. Prev. Alzheimer Dis..

[179] Colini Baldeschi A., Zattoni M., Vanni S., Nikolic L., Ferracin C., La Sala G., Summa M., Bertorelli R., Bertozzi S.M., Giachin G., Carloni P., Bolognesi M.L., De Vivo M., Legname G. (2022 Jul 14). Innovative non-PrP-targeted drug strategy designed to enhance prion clearance. J. Med. Chem..

[180] Staderini M., Vanni S., Baldeschi A.C., Giachin G., Zattoni M., Celauro L., Ferracin C., Bistaffa E., Moda F., Pérez D.I., Martínez A., Martín M.A., Martín-Cámara O., Cores Á., Bianchini G., Kammerer R., Menéndez J.C., Legname G., Bolognesi M.L. (2023). Bifunctional carbazole derivatives for simultaneous therapy and fluorescence imaging in prion disease murine cell models. Eur. J. Med. Chem..

[181] Roberts B.E., Duennwald M.L., Wang H., Chung C., Lopreiato N.P., Sweeny E.A., Knight M.N., Shorter J. (2009). A synergistic small-molecule combination directly eradicates diverse prion strain structures. Nat. Chem. Biol..

[182] Lucchetti J., Fracasso C., Balducci C., Passoni A., Forloni G., Salmona M., Gobbi M. (2019). Plasma and brain concentrations of doxycycline after single and repeated doses in wild-type and APP23 mice. J. Pharmacol. Exp. Ther..

